# Astilbin prevents osteoarthritis development through the TLR4/MD‐2 pathway

**DOI:** 10.1111/jcmm.15915

**Published:** 2020-10-16

**Authors:** Shuaibo Sun, Zijian Yan, Xiaolong Shui, Weihui Qi, Yanlin Chen, Xinxian Xu, Yuezheng Hu, Weijun Guo, Ping Shang

**Affiliations:** ^1^ Department of Orthopaedic Surgery The Second Affiliated Hospital and Yuying Children’s Hospital of Wenzhou Medical University Wenzhou China; ^2^ Department of Rehabilitation The Second Affiliated Hospital and Yuying Children’s Hospital of Wenzhou Medical University Wenzhou China

**Keywords:** astilbin, osteoarthritis, TLR4/MD‐2

## Abstract

Osteoarthritis has become one of the main diseases affecting the life of many elderly people with high incidence of disability, and local chronic inflammation in the joint cavity is the most crucial pathological feature of osteoarthritis. Astilbin is the main active component in a variety of natural plants such as Hypericum perforatum and Sarcandra glabra, which possess antioxidant and anti‐inflammatory effects. At present, there is no study about the protective effect of Astilbin for osteoarthritis. The purpose of this study was to investigate the effect of Astilbin in human OA chondrocytes and mouse OA model, which was established by surgery‐mediated destabilization of the medial meniscus (DMM). In vitro, we found that Astilbin pre‐treatment inhibited lipopolysaccharide (LPS)‐induced overproduction of inflammation‐correlated cytokines such as nitric oxide (NO), prostaglandin E2 (PGE2), tumour necrosis factor α (TNF‐α) and interleukin 6 (IL‐6), and suppressed overexpression of inflammatory enzymes such as inducible nitric oxide synthase (iNOS) and cyclooxygenase 2 (COX‐2). Astilbin, on the other hand, prevented the LPS‐induced degradation of extracellular matrix (ECM) by down‐regulating MMP13 (matrix metalloproteinases 13) and ADAMTS5 (a disintegrin and metalloproteinase with thrombospondin motifs 5). Moreover, by inhibiting the formation of the TLR4/MD‐2/LPS complex, Astilbin blocked LPS‐induced activation of TLR4/NF‐κB signalling cascade. In vivo, Astilbin showed the chondro‐protective effect in the surgical‐induced OA mouse models. In conclusion, our findings provided evidence that develops Astilbin as a potential therapeutic drug for OA patients.

## 1 INTRODUCTION

Osteoarthritis (OA) is a degenerative joint disease with substantial morbidity most common among older people. At present, there are 120 million OA patients in China.^1^, ^2^ The aetiologic agent of OA is related to age, obesity, gender and other factors.^3^, ^4^ There is currently no effective cure for OA.^1^ Previous studies report that inflammation plays a crucial role in the pathogenesis of OA.^5^, ^6^, ^7^ Kraus et al reported the presence of lipopolysaccharide (LPS) and lipopolysaccharide‐binding protein (LBP) in serum and synovial fluid of OA patients.^8^ LPS is known to cause low‐grade inflammation and accelerate OA development.^9^, ^10^ Therefore, in this study, LPS was used to mimic OA in cell experiments.

Toll‐like receptors (TLRs), are cell membrane receptors, participate in the progression of OA by activating a variety of downstream signalling.^11^, ^12^ Mild TLRs activation triggers a self‐protective mechanism, but excessive activation of TLRs contributes to inflammatory responses due to the continuous secretion of pro‐inflammatory cytokines and chemokines. Although there are many subtypes of TLRs, chondrocytes mainly express TLR4, which is reported to be involved in OA progression.^10^, ^13^, ^14^, ^15^ As an effective promoter of OA‐related disease‐associated molecular patterns (DAMPs), LPS initiates a series of inflammatory reactions by activating TLR4 and subsequently leads to structural damage of joint cartilage. Myeloid differentiation protein‐2 (MD‐2) is the extracellular domain of TLR4, which recognizes LPS and assists it to bind TLR4.^16^ After the LPS/TLR4/MD‐2 complex is formed, downstream proteins such as myeloid differentiation factor 88 (MyD88), interleukin‐1 (IL‐1) receptor‐related kinase (IRAKs) and tumour necrosis factor (TNF) receptor‐related factor 6 (TRAF6) bind to the complex and further activate inflammation‐related signalling pathways including the NF‐κB pathway and eventually leading to the occurrence of inflammation and ECM catabolism.^12^, ^17^, ^18^ Therefore, targeted inhibition of the TLR4/MD‐2 pathway is a potentially promising therapy to prevent OA development.

Astilbin is a flavonoid widely found in daily diets and traditional medicines such as Dimorphandra Mollis, grape, Smilax corbularia, Hypericum perforatum, Sarcandra glabra and other natural plants.^19^, ^20^, ^21^ Modern pharmacological research shows that Astilbin has anti‐oxidation,^22^ anti‐inflammatory,^23^, ^24^ anti‐tumour^25^ and other pharmacological activities. It is reported that Astilbin can alleviate LPS‐induced inflammatory response in macrophages^24^ and suppress high glucose‐induced inflammation in rat glomerular mesangial cells by intervening TLR4/MyD88/ NF‐κB axis.^26^ However, the effect of Astilbin on osteoarthritis is not clear. Therefore, in this study, we investigated the anti‐inflammatory effect of Astilbin in LPS‐induced human chondrocytes and its protective effect and underlying mechanism in the surgery‐induced mouse OA model.

## 2 MATERIALS AND METHODS

### 2.1 Chemicals and reagents

Astilbin (purity > 98%) was purchased from BOC Sciences (New York, USA). Cell Counting Kit‐8 (CCK‐8) was purchased from Dojindo (Kumamoto, Japan). LPS, type II collagenase and dimethylsulphoxide (DMSO) were obtained from Sigma‐Aldrich (St Louis, MO, USA). The primary antibodies against TLR4, MyD88, TRAF6, Lamin B1 and GADPH were obtained from Abcam (Cambridge, UK); iNOS antibodies were obtained from Sigma‐Aldrich (St Louis, MO, USA); and MD2 antibody was purchased from eBioscience (eBioscience, SanDiego, CA). Antibodies against COX‐2, p65 and IκBα were purchased from Cell Signaling Technology (Danvers, MA, USA); anti‐IRAK1, goat anti‐rabbit and antimouse IgG‐HRP were from Bioworld (OH, USA). Recombinant human MD2 and TLR4 protein (rhMD2 and rhTLR4) were purchased from R&D Systems (Minneapolis, MN, USA). Biotin‐labelled LPS (Biotin‐LPS) was obtained from InvivoGen (San Diego, CA, USA). Alexa Fluor^®^488 labelled and Alexa Fluor^®^594 labelled Goat Anti‐Rabbit IgG (H + L) the second antibody were purchased from Jackson ImmunoResearch (West Grove, PA, USA). 4′,6‐Diamidino‐2‐phenylindole (DAPI) was purchased from Beyotime (Shanghai, China), and cell culture reagents were obtained from Gibco (Grand Island, NY, USA).

### 2.2 Primary human chondrocyte culture

The human articular cartilage sample collection was approved by the medical ethics committee of the Second Affiliated Hospital of Wenzhou Medical University (ethics standard: LCKY‐2017‐25) and followed the guidelines of Helsinki Declaration.^27^ The cartilage tissues were obtained from six patients (58‐69 years old, 3 males and 3 females) who underwent total knee arthroplasty, and informed consent was obtained from all patients before the start of the study. To ensure that full‐thickness cartilage was obtained, the cartilage tissue was cut at the posterior femoral condyles and avoided selecting cartilage tissue with a damaged surface. The cartilage tissues were cut into 1.0 × 1.0 × 1.0 mm^3^ pieces and PBS was used to blow and wash the tissues repeatedly. The cartilage slices were treated with 2mg/ml type II collagenase and digested for 4h in Dulbecco’s modified Eagle's medium (DMEM)/F12 at 37°C. Following the basic digestion of the tissue, centrifugation at 1000 rmp for 5 minutes was done, and the supernatant discarded. The chondrocytes were cultured at a density of 2 × 10^5^ cells/ml in a 6‐well plate in DMEM/F12 supplemented with 10% FBS and 1% antibiotics and incubated at 37°C with 5% CO_2_. The morphology and the adherence of the cells were observed after one day. The media was changed every 2‐3 days, and the cells passaged after attaining 70%‐90% confluence. To avoid changes in cell phenotype, only the first to third generations of chondrocytes were considered for these experiments.

### 2.3 Animal model

A total of forty‐five 8‐week‐old C57BL/6 male wild‐type (WT) mice were purchased from the Shanghai animal research centre of the Chinese Academy of Sciences and were randomly divided into three groups (n = 15). The animal protection and use regulations were performed in accordance with the guidelines for the protection and use of experimental animals issued by the National Institutes of Health and approved by the animal protection and use Committee of Wenzhou Medical University (code of Ethics: wydw2014‐0127). The mice were raised in a relatively suitable environment (temperature 20‐28 ℃, humidity 45%‐70%, 24‐hour cycle light, free food, and drinking water). Subsequently, the experimental mice underwent surgical destabilization of the medial meniscus (DMM) to induce OA.^28^ Briefly, after intraperitoneal anaesthesia with 2% (w/V) pentobarbital (40 mg/kg), the skin and joint capsule of the right knee were cut along the medial side of patellar ligament using a mini blade, and the muscle and fat pad separated to expose the joint cavity and medial meniscus. The ligament of the medial meniscus was cut off using a knife or scissors. Finally, the tissue and skin were sutured layer by layer. Following intraperitoneal anaesthesia, the sham operation was performed on the right knee joint of the sham group mice, the skin and joint capsule were cut along the medial patellar ligament, and then, the wound was sutured. After the operation, penicillin was injected to prevent infection.

### 2.4 Experimental design

To evaluate the protective effect of different concentrations of Astilbin in vitro, cells were treated with 1 μg/mL LPS alone or in combination with different concentrations (10, 20 or 40 μmol/L) of Astilbin pre‐treatment. The control group was not treated except for the change of culture medium. After 24 hours of culture, the cells were harvested. To study the activation of NF‐κB and TLR4, the duration of LPS stimulation was 2 hours. Meanwhile, to study functional changes such as inflammation or ECM markers, the duration was extended to 24 hours.

For in vivo experiments, the mice underwent surgical destabilization of the medial meniscus (DMM) as described above. After DMM, the mice in the Astilbin group were administered with Astilbin (20 mg/kg/d) by gastric perfusion once a day for 8 weeks (Astilbin diluted in 0.5% CMC). Similarly, the mice in the DMM alone group received a similar amount of saline. All the animals were killed 8 weeks after the operation, and the cartilage tissue samples collected for imaging and histological analysis.

### 2.5 Cell viability

Cell viability was assessed using the CCK‐8 kit according to the manufacturer's instructions. Briefly, human OA chondrocytes were incubated in 96‐well plates at a density of 5 × 10^3^ cells/cm^2^ for 24 hours. The cells were treated with different concentrations of Astilbin (0, 5, 10, 20, 40, 80 μmol/L) for 24 or 48 hours. After washing the cells with phosphate buffered saline (PBS), 100 μL DMEM/F12 containing 10% CCK‐8 solution was added to each well and incubated at 37°C for 4 hours. A microplate reader (Thermo Fisher, Waltham, MA, USA) was used to measure the absorbance at 450 nm. All experiments were conducted five times.

### 2.6 Griess reaction and ELISA

The light absorption value of the sample was detected under the ultraviolet spectrophotometer, and the NO content of the corresponding sample was calculated according to the manufacturer's instructions. The blood samples collected from the abdominal aorta were centrifuged for 15 minutes at 1000 *g*. The concentration of TNF‐α, PGE2, IL‐6, Collagen II, aggrecan, ADAMTS‐5 and MMP13 in each sample was detected using commercial ELISA kits (R&D Systems, Minneapolis, MN) according to the manufacturer's instructions. All tests were conducted five times.

### 2.7 Protein extraction

Total proteins were extracted from chondrocytes using RIPA lysis buffer. Cytoplasmic and nuclear proteins were extracted using the corresponding protein extraction kit (Beyotime, Shanghai, China). The sample solution was lysed on ice by ultrasound and centrifuged at 15000 x*g* at 4°C for 30 minutes. The concentration of the protein was determined using the BCA protein assay kit (Beyotime, Shanghai, China).

### 2.8 Western blotting

A similar amount of protein (40 μg) was separated by sodium dodecyl sulphate‐polyacrylamide gel electrophoresis (SDS‐PAGE) and transferred to a polyvinylidene difluoride membrane (Bio‐Rad, USA). After blocking with 5% skim milk for 2 hours and washing 3 times with TBST, the membranes were incubated with primary antibodies against iNOS (1:1000), MD‐2 (1:500), COX‐2 (1:1000), p65 (1:1000), TLR4 (1:500), IκBα (1:1000), TRAF6 (1:1000), MyD88 (1:1000), IRAK1 (1:500), GAPDH (1:5000) and Lamin B (1:5000) at 4°C overnight. The membranes were then incubated with the corresponding secondary antibody at room temperature for 2 hours. After washing with TBST 3 times, antibody binding was visualized by Electrochemiluminescence Plus reagent (Invitrogen), and the Image Lab 3.0 software (Bio‐Rad) used to quantitatively analyse the intensity of the bands.

### 2.9 MD‐2 and TLR4 competitive ELISA assay

Human MD‐2 or TLR4 antibody was coated on 96‐well plates using 10 mmol/L Tris‐HCl buffer (pH 7.5). The plates were incubated at 4 ℃ overnight, washed thrice with poly (butylene succinate‐*co*‐butylene terephthalate) PBST, followed by blocking with 3% bovine serum albumin at room temperature for 1.5 hours. After blocking, 4 μg/mL of recombinant human MD‐2 (rhMD‐2) or recombinant Toll‐like receptor 4 (rhTLR4) was added to 10 mmol/L Tris‐HCl buffer (pH 7.5), and the plates were further incubated at room temperature for 1.5 hours. After washing with PBST, biotin‐LPS and different concentrations of Astilbin (0, 10, 20 or 40 μmol/L) were added at room temperature for 1.5 hours. The horseradish peroxidase combined with streptavidin (Beyotime, Shanghai, China) was added at room temperature and incubated for 1 hour. After the addition of tetramethylbenzidine substrate (eBioscience, San Diego, CA, USA), horseradish peroxidase activity was then measured at 450 nm using a SpectraMax M5 plate reader (Molecular Devices, Sunnyvale, CA, USA).

### 2.10 Immunofluorescence

For collagen II and MMP‐13 staining, chondrocytes were placed in a 6‐well plate and treated with 1 μg/mL LPS alone or 1 μg/mL LPS containing 40 μmol/L Astilbin for 24 hours and incubated overnight in a serum‐starved medium. For p65 staining, the treatment time for LPS and Astilbin was 2 hours. The samples were rinsed thrice in PBS, fixed with 4% paraformaldehyde for 15 minutes and permeabilized in PBS containing 0.5% Triton X‐100 at room temperature for 15 minutes. The cells were then covered with 5% goat serum at room temperature for 1 hour, washed with PBS and then incubated with primary antibodies against collagen II (1:200), MMP‐13 (1:200) and p65 (1:200) at 4°C overnight. After washing thrice with PBS, the glass plates were incubated with Alexa Fluor^®^488‐labelled or Alexa Fluor^®^594‐conjugated secondary antibodies (1:400) at room temperature for 1 hour and labelled with DAPI (Invitrogen) for 1 minute. Finally, five fields of each slide were randomly selected for analysis using fluorescence microscopy (Olympus, Tokyo, Japan).

### 2.11 Immunoprecipitation

Cell lysates were obtained as mentioned above, incubated with sufficient anti‐TLR4 antibody for 1h, and protein G‐Sepharose beads added to retrieve the immune complexes overnight at 4°C in a shaking bed. The precipitates were washed with cold PBS for 4 times and boiled in sample buffer to release the proteins. Similarly, the level of MD‐2 was further detected by immunoblotting with the anti‐MD‐2 antibody (IB).

### 2.12 Molecular modelling

ChemBioDraw software was used to draw the molecular structure of Astilbin, and the energy of Astilbin minimized using ChemBio3D. Access to the Protein Data Bank (https://www.rcsb.org/) to obtain the crystal structure of the human MD2/lipid IVa complex (PDB code 2E59) was done. After minimizing the energy with PyMOL (version 1.7.6), the lowest energy conformation for docking was determined using default parameters. To complete the protein‐ligand docking analysis, AutoDock Tools (version 1.5.6) was used. Finally, UCSF PyMOL was used to create the image of a 3D view.

### 2.13 X‐ray imaging method

After 8 weeks of operation, all animals within or without treatment were received X‐ray examination. A digital X‐ray machine (Kubtec Model XPERT.8; KUB Technologies Inc) was used to assess changes in knee joint space, osteophyte formation and cartilage surface calcification. Appropriate images are obtained using the following settings: 50kV and 160 μA.

### 2.14 Histological analysis

The knee joint specimens were fixed with 4% paraformaldehyde for 24 hours at 4°C, and decalcified with 10% EDTA solution for 4 weeks at 4°C. Then, dehydrate with an alcohol gradient, and embedded in paraffin blocks. Continuous 5‐μm‐thick sagittal sections (about 100) were cut across the entire medial compartment of the knee joint until anterior cruciate ligament junction. For each joint, we chose one pieces every 50 μm, a total of 10 pieces, for safranin O/fast green staining to assess the cartilage damage. The stained parts were photographed digitally under a microscope. The destruction of articular cartilage was evaluated by the Osteoarthritis Research Society International (OARSI) scoring system for the medial tibial condyle and medial femoral plateau.^29^ Sections were divided as follows (6 OA grades): 1 = surface fibrillation without cartilage loss, 2 = vertical cracks and loss of surface layer (any percentage or joint surface area), 3 = 1%‐25% of quadrant width of vertical cracks/erosion to the calcified layer lesion, 4 = lesion reaches of 25%‐50% of quadrant width of the calcified cartilage, 5 = lesion reaches of 50%‐75% of quadrant width of the calcified cartilage, and 6 = lesion reaches of >75% of quadrant width of the calcified cartilage. Then, the extent of articular cartilage destruction was graded by the summed OARSI scores (0‐12) from the medial femoral condyle and medial tibial plateau.

### 2.15 Statistical analysis

Each experiment was repeated at least 5 times. The results were expressed as the mean ± standard deviation, and SPSS 20.0 statistical software was used to process all data of the experiment. Comparisons between control and treatment groups were analysed using Mann‐Whitney U test. The difference was statistically significant when *P* < 0.05.

## 3 RESULTS

### 3.1 Effect of Astilbin on human chondrocyte viability

**Figure 1:**
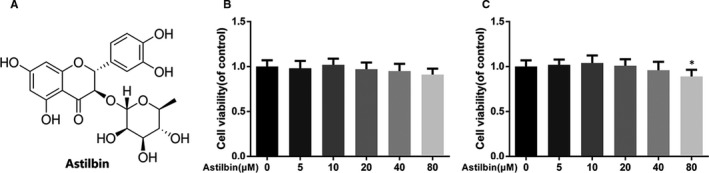
Effect of Astilbin on human chondrocytes viability. A, Chemical structure of Astilbin. (B,C) Cytotoxicity of Astilbin on chondrocytes at 24 h and 48 h. The data in the figures represent the averages ± SD. Significant differences among different groups are indicated as *P < 0.05 vs control group, n = 5

The molecular structure of Astilbin is shown in Figure 1A. We employed the CCK‐8 assay to evaluate the cytotoxicity of Astilbin, and chondrocytes were cultured with different concentrations of Astilbin (0, 5, 10, 20, 40 and 80 μmol/L) for 24 or 48 hours, followed by cholecystokinin‐octapeptide (CCK‐8) assay. The results are shown in Figure 1B,C, after 24 hours, where Astilbin showed no cytotoxicity to human OA chondrocytes. However, after 48 hours, Astilbin showed cytotoxicity to human OA chondrocytes at a concentration of 80 μmol/L. Therefore, the Astilbin doses used in subsequent experiments were 10, 20 and 40 μmol/L.

### 3.2 Effect of Astilbin on the expression of iNOS, COX‐2, PGE2, NO, TNF‐α and IL‐6 in human osteoarthritis chondrocytes induced by LPS

**Figure 2:**
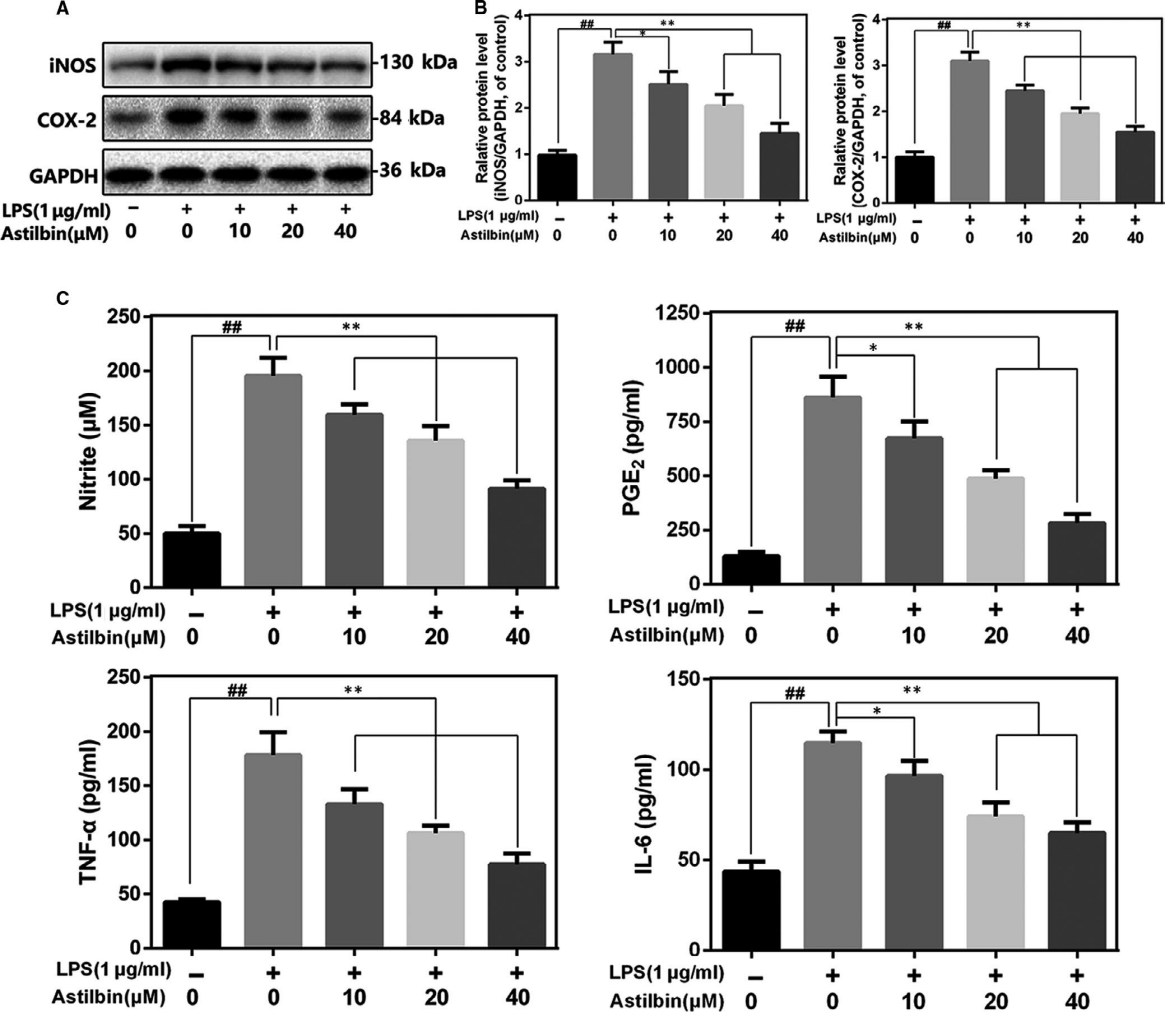
Influence of Astilbin in LPS‐induced inflammatory reaction in human chondrocytes. (A,B) iNOS and COX‐2 protein level in chondrocytes measured by Western blot. C, Effect of Astilbin on LPS‐exposed IL‐6, TNF‐α, PGE2 and NO production in human chondrocytes. The data in the figures represent the averages ± SD. Significant differences among different groups are indicated as ^##^ *P* < 0.01, vs control group; **P < 0.01 vs LPS alone treatment group, n = 5

To determine the effect of Astilbin on LPS‐induced inflammatory response in human OA chondrocytes, the expression levels of several inflammatory markers were determined. As shown in Figure 2A,B, LPS‐induced overexpression of COX‐2 and iNOS was inhibited by Astilbin treatment in a dose‐dependent manner (10, 20 and 40 μmol/L). As shown in Figure 2C, ELISA results showed that LPS up‐regulated the expression of endogenous NO, PGE2, TNF‐α and IL‐6 in chondrocytes. However, following treatment with Astilbin, the expression decreased in a dose‐dependent manner. These data indicated that Astilbin inhibits the production of these inflammatory cytokines in a dose‐dependent manner.

### 3.3 Effect of Astilbin on LPS‐induced extracellular matrix (ECM) degradation in human OA chondrocytes

**Figure 3:**
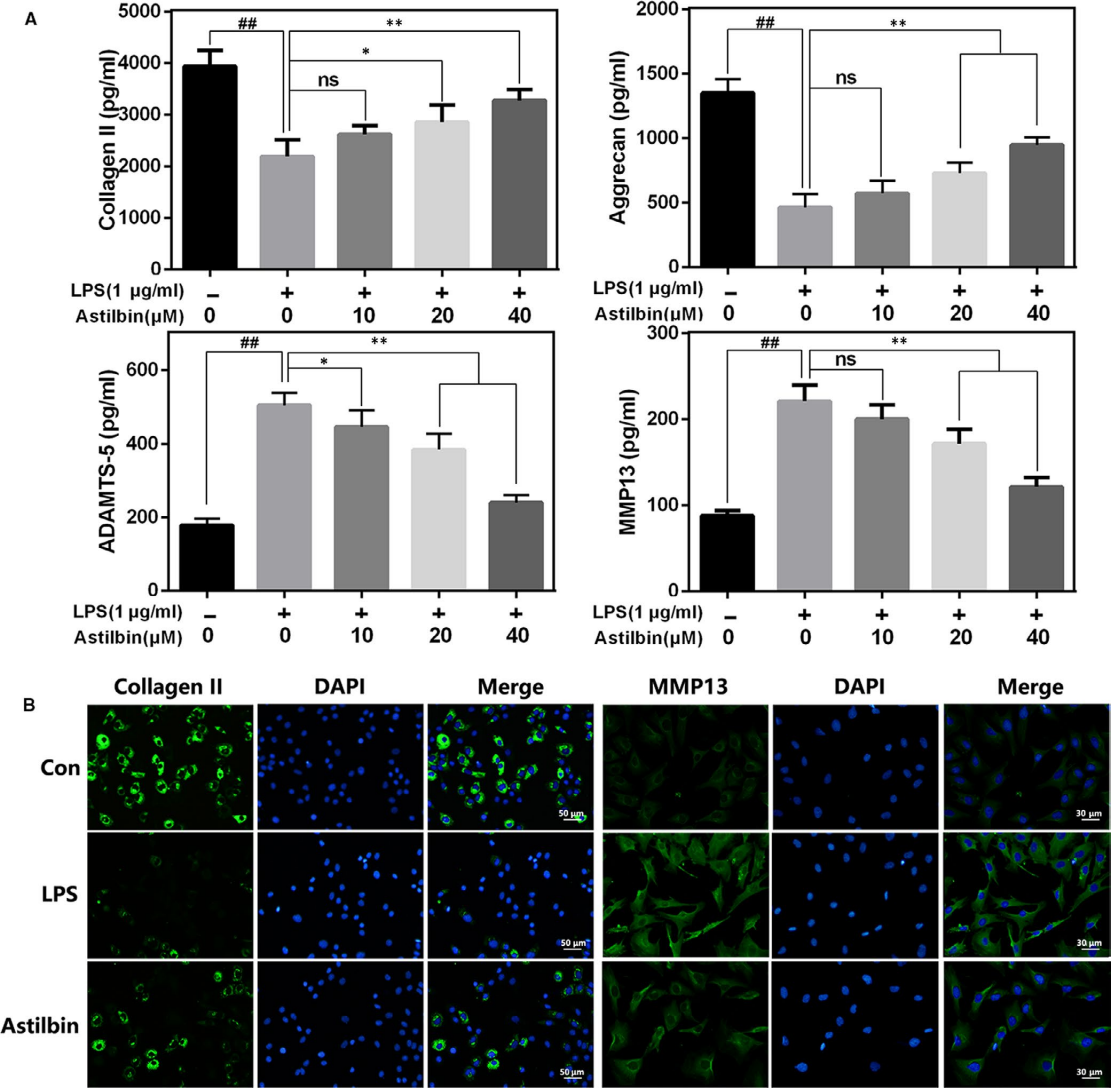
Influence of Astilbin in LPS‐mediated ECM degeneration in human chondrocytes. A, The level of Collagen II, aggrecan, MMP13 and ADAMTS‐5 in chondrocytes treated as above was visualized by ELISA. B, The representative collagen II and MMP13 were detected by the immunofluorescence combined with DAPI staining for nuclei (scale bar: 50 µm or 30 µm). The data in the figures represent the averages ± SD. Significant differences among different groups are indicated as ^##^ *P* < 0.01, vs control group; *P < 0.05, **P < 0.01 vs LPS alone treatment group, n = 5

The effect of Astilbin on the degradation of collagen II and aggregates induced by LPS in human OA chondrocytes was investigated. Compared with LPS alone group, Astilbin treatment increased the expression of aggrecan and collagen II and suppressed the production of matrix metalloproteinases (MMP)‐13 and ADAMTS‐5 in a dose‐dependent manner (Figure 3A). Additionally, the results of immunofluorescence staining of collagen II and MMP13 were consistent with ELISA results (Figure 3B). Taken together, these results indicated that Astilbin alleviates LPS‐mediated ECM degradation in human OA chondrocytes.

### 3.4 Effect of Astilbin on LPS‐induced NF‐κB activation in human OA chondrocytes

**Figure 4:**
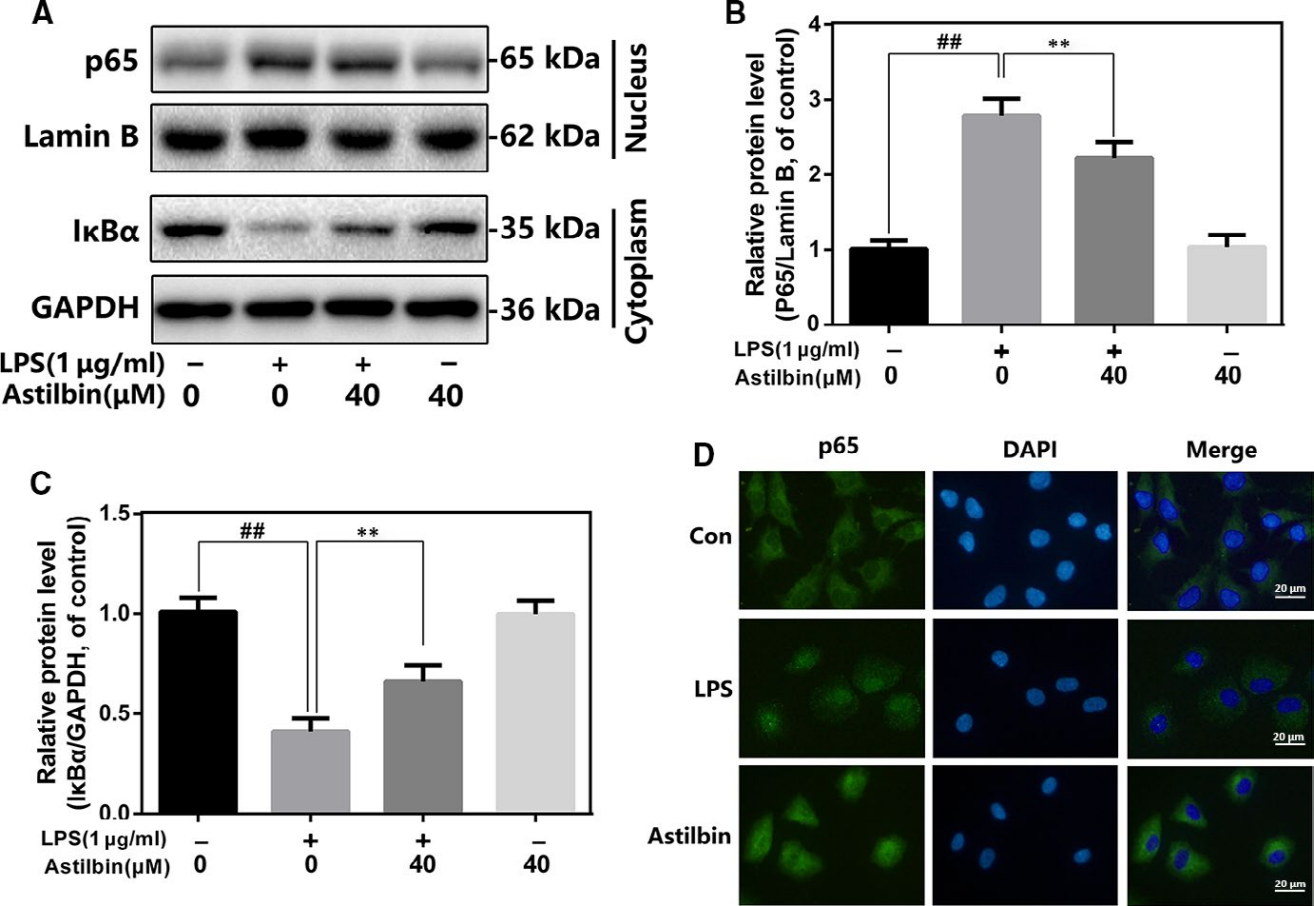
Influence of Astilbin in LPS‐mediated activation of NF‐κB pathway in human chondrocytes. (A,C,D) The protein expressions of IκBα in cytoplasm and p65 in nuclear in chondrocytes treated as above were detected by Western blot. B, The nuclei translocation of p65 was detected by the immunofluorescence combined with DAPI staining for nuclei (scale bar: 20 µm). The data in the figures represent the averages ± SD. Significant differences among different groups are indicated as ^##^ *P* < 0.01, vs control group; **P < 0.01 vs LPS alone treatment group, n = 5

To determine the anti‐inflammatory mechanism of Astilbin, we assessed the change of NF‐κB signalling activity. We found that LPS stimulated IκBα degradation in the cytoplasm and increased p65 content in the nucleus. However, Astilbin pre‐treatment (40 μmol/L) inhibited this phenomenon, and the NF‐κB activity in the Astilbin‐alone group did not change (Figure 4A,B, and C). Subsequently, we examined the p65 translocation in human OA chondrocytes induced by LPS. Immunofluorescence staining in. 4D shows that the p65 positive spot in the LPS‐stimulated group was translocated from the cytoplasm to the nucleus compared with the control group. However, pre‐treatment with Astilbin (40 μmol/L) sharply reduced the translocation of p65. These results indicated that Astilbin could inhibit NF‐κB signalling activity in LPS‐exposed human OA chondrocytes.

### 3.5 Effects of Astilbin on the interaction of TLR4 and MD‐2 in LPS‐induced human OA chondrocytes

**Figure 5:**
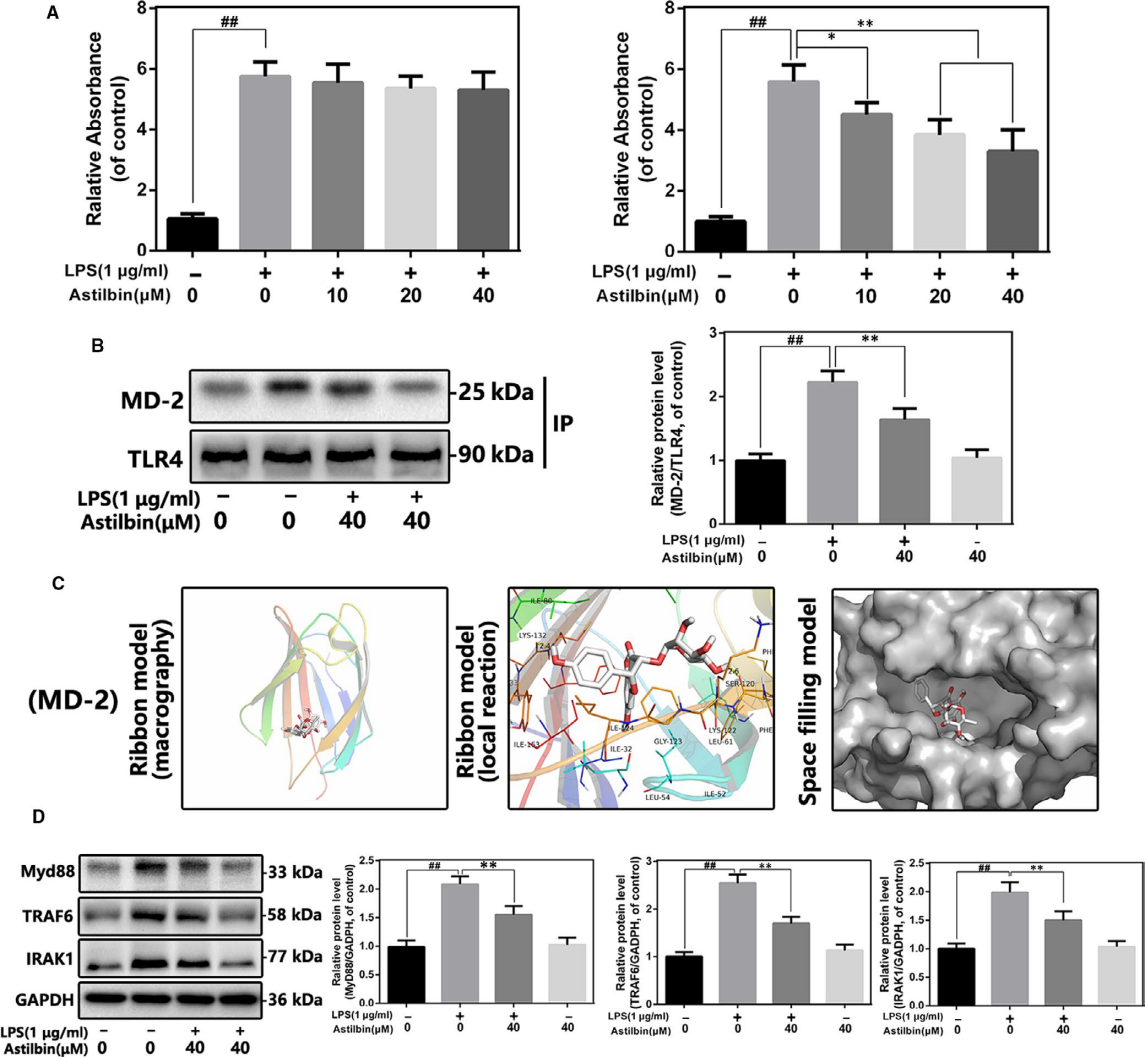
Influence of Astilbin on LPS‐induced TLR4/MD‐2 signalling activation. A, The binding of biotin‐labelled LPS to rhMD‐2 and rhTLR4 was examined by competitive ELISA. B, The complexes of TLR4‐MD‐2 in chondrocytes treated as above were detected by immunoprecipitation. C, Astilbin was docked with the MD‐2 structure. Docking studies were performed as described in Materials and methods. The protein residues are shown in a ribbon model. The proposed binding pose of Astilbin shows interactions with LYS‐132. The space‐filling models show the binding of Astilbin in the inhibitory binding pockets. D, The protein expressions of MyD88, IRAK‐1 and TRAF‐6 in chondrocytes treated as above were detected by Western blot. The data in the figures represent the averages ± SD. Significant differences among different groups are indicated as ^##^ *P* < 0.01, vs control group; **P < 0.01 vs LPS alone treatment group, n = 5

Accumulating evidence demonstrates that LPS binds to TLR4 by recognizing the adaptor protein MD‐2. We first used competitive ELISA to explore the effect of Astilbin in the TLR4/MD‐2 axis, which is an upstream molecule of NF‐κB signalling. As shown in Figure 5A, Astilbin reduced the binding of LPS to rhMD‐2, but not to rhTLR4, which indicated that Astilbin acts on the TLR4/MD‐2 complex by inhibiting the binding of LPS to MD‐2 and not through any interaction with TLR4. Subsequently, we carried out co‐immunoprecipitation experiments (Figure 5B). LPS was found to promote the binding of TLR4 and MD‐2, whereas the addition of Astilbin (40 μmol/L) inhibited the formation of the TLR4/MD‐2 complex. Moreover, the interaction between TLR4 and MD‐2 did not change when treated with Astilbin alone, indicating that Astilbin competitively occupied the LPS binding site of the TLR4/MD‐2 complex. Molecular docking analysis results in Figure 5C shows that Astilbin has a high affinity for the hydrophobic position of MD‐2 (−6.5 kcal/mol), and a hydrogen bond is formed between Astilbin and the LYS‐132 residue of MD‐2 by a band model. The space‐filling model showed that Astilbin is completely embedded in the inhibition cavity of MD‐2. Taken together, these results suggested that Astilbin could competitively inhibit the binding of LPS to MD‐2, thereby preventing the formation of the TLR4/MD‐2 complexes.

### 3.6 Effects of Astilbin on LPS‐induced activation of Toll adapters in human OA chondrocytes

LPS induced TLR4 signalling activation by recognizing MD‐2 and up‐regulating several Toll adapters, such as MyD88, IRAK‐1 and TRAF‐6, which play a key role in NF‐κB‐mediated inflammation. To determine the effect of Astilbin on these signal cascades, Western blot analysis was used to evaluate the expression levels of MyD88, IRAK‐1 and TRAF‐6 in LPS‐induced human OA chondrocytes. As shown in Figure 5D, LPS promoted the expression of MyD88, IRAK‐1 and TRAF‐6, whereas this phenomenon was reversed by Astilbin pre‐treatment.

### 3.7 Astilbin ameliorates OA development in the mouse model of DMM

**Figure 6:**
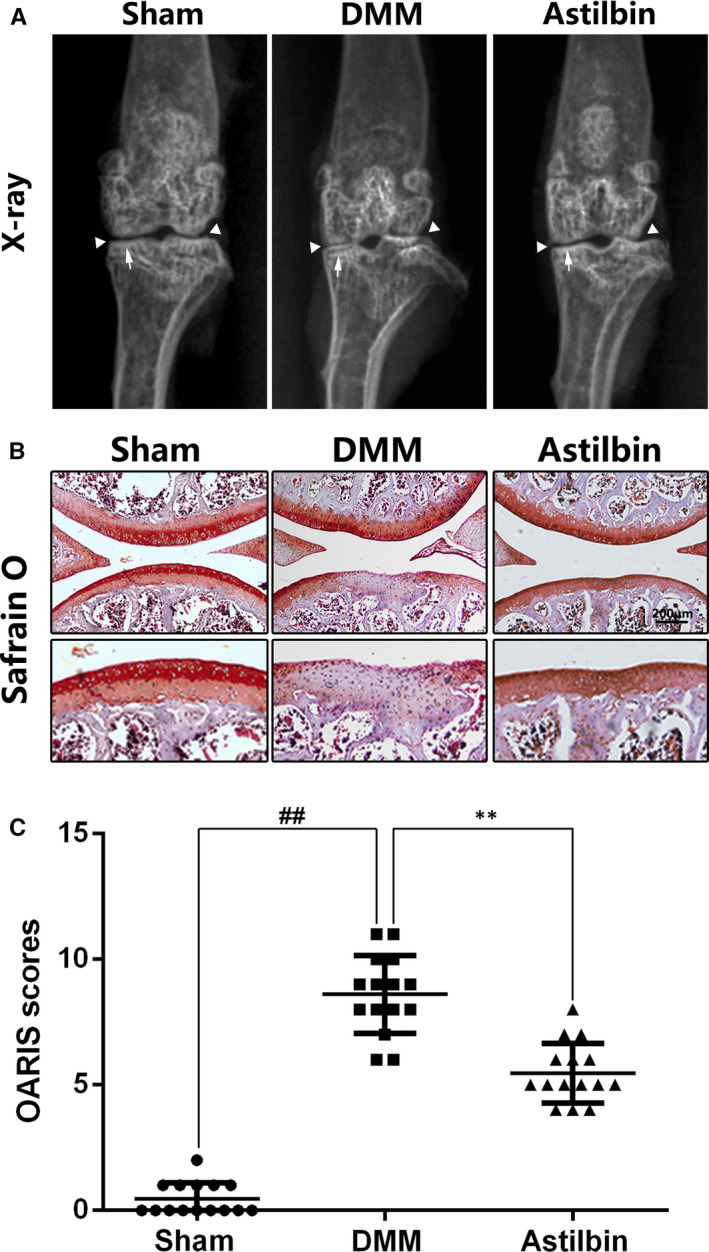
Astilbin attenuates OA development in vivo. A, Digital X‐ray image of mouse knee joints from different experimental groups. Narrowing of joint space was found in both OA and treatment group (white triangles), the calcification of cartilage surface was obviously shown in OA group (white arrows). B, Representative S‐O staining of cartilage from different experimental groups at 8 wk post‐surgery (scale bar: 200 µm). C, Diagrams showed the OARIS socres of cartilage. The data in the figures represent the averages ± SD. Significant differences among different groups are indicated as ^##^ *P* < 0.01, **P < 0.01, n = 15

To study the protective ability of Astilbin in the OA process in vivo, the DMM model was established by surgery. Mice in the Astilbin group received 40 mg/kg of Astilbin orally once a day for 8 weeks and were then sacrificed for imaging and histological analysis. As shown on the radiographs in Figure 6A, the surface density of cartilage in the DMM group was higher compared with the sham group, and the joint space was severely narrowed. However, in the Astilbin treatment group, although the joint space was narrowed, the surface calcification of cartilage was lighter. Histological analysis of OA was performed by Safranin O staining (Figure 6B) and quantified using the Osteoarthritis Research Society International (OARSI) scoring system. We found that the cartilage surface in the sham group was smooth and stained red. In contrast, cartilage wear and erosion were observed in the DMM group. The degree of cartilage erosion in the Astilbin treatment group was lower compared with the DMM group. The OARSI score (Figure 6C) of the DMM group was significantly higher than that of the sham group, which was consistent with the Safranin O staining. However, the OARSI score in the Astilbin group was lower compared with the DMM group. In conclusion, these results suggested that Astilbin delays the development of OA in vivo.

## 4 DISCUSSION

Osteoarthritis is a common clinical degenerative joint disease with increasing morbidity rate.^1^, ^2^, ^3^ Unfortunately, the aetiology of OA is complex and remains unclear.^3^, ^30^ Although non‐steroidal anti‐inflammatory drugs (NSAIDs) can effectively alleviate the pain in patients, they also have obvious limitations, such as intestinal adverse reactions, and do not delay the progression of OA.^31^, ^32^, ^33^ Therefore, it is urgent to explore drugs, which can prevent and delay the progression of OA.^3^, ^34^

Accumulating evidence demonstrates that inflammation is closely related to the development of OA.^35^, ^36^ LPS has been widely used as an agent to stimulate the inflammation and apoptosis in chondrocytes in vitro.^9^, ^37^ In previous studies, the dosage of LPS varying from 0.1 to 50 μg/mL was applied to chondrocytes, a high concentration of LPS was used to study the mechanism of cell death, whereas the low dosage of LPS was considered more suitable for mimicking the inflammatory environment.^37^, ^38^, ^39^ In this study, we used LPS at 1 μg/mL for cellular experiments.

Astilbin is a flavonoid extracted, separated and identified from Dimorphandra Mollis, grape, Smilax corbularia, Hypericum perforatum, Sarcandra glabra and other natural plants.^19^, ^20^, ^21^ Modern pharmacology research shows that it possesses anti‐oxidation,^22^ anti‐inflammatory,^23^, ^24^ anti‐tumour^25^ and other pharmacological activities. However, the mechanism of protection in OA remains unclear. Therefore, in this study, we studied the anti‐inflammatory effect of Astilbin in OA.

Previous studies have reported that the progression of OA is mainly caused by several pro‐inflammatory mediators, iNOS catalyses NO or COX‐2 to induce PEG2 production, then increases the content of MMPs and ADAMTs, finally promoting the degradation of collagen II and proteoglycan.^40^, ^41^ Moreover, TNF‐α and IL‐6 also participate in the development of OA.^42^ In this study, we found that Astilbin treatment inhibited the LPS‐induced increase in PGE2 and NO in human OA chondrocytes and up‐regulation of COX‐2 and iNOS, as well as TNF‐α and IL‐6. Besides its anti‐inflammatory effects, Astilbin also inhibits ECM degradation. Following LPS stimulation, the synthesis and secretion of MMP‐13 and ADAMTS‐5 increased in human OA chondrocytes, and the expression of collagen II and aggrecan decreased, and these phenomena were reversed after Astilbin pre‐treatment. These results suggest that Astilbin has a potential therapeutic role in preventing OA progression.

To explore the protective mechanism of Astilbin during OA, the effect of Astilbin on the NF‐κB pathway was studied, which is a crucial inflammatory signalling pathway for OA.^43^, ^44^ The results showed that Astilbin inhibited LPS‐induced NF‐κB activation in human chondrocytes. However, even though we have confirmed that Astilbin can exert anti‐inflammatory effects by targeting NF‐κB, its potential upstream cascade effect also requires to be studied further. Previous studies reveal that TLR4 signalling is currently one of the most widely studied upstream cascades, and the concentration of TLR4 in NF‐κB activation and OA development has received increasing attention.^16^, ^45^ TLR4‐targeted antagonist is emerging as a new drug to alleviate the progression of OA. Therefore, in this study, we investigated whether Astilbin affects the TLR4/MD‐2 axis using competitive ELISA. Astilbin was found to prevent the binding between LPS and MD‐2 but showed no significant effect on TLR4. Co‐immunoprecipitation results showed that the interaction between TLR4 and MD‐2 was greatly reduced by Astilbin treatment. Docking analysis simulated the underlying mechanism of Astilbin acting on MD‐2 and showed that Astilbin specifically binds the MD‐2 inhibitory pocket and interferes with the formation of the TLR4/MD‐2 complex. These results suggest that Astilbin may affect the TLR4/MD‐2 axis by competitively binding to MD‐2. When the TLR4/MD‐2/LPS complex is formed, the intracellular domains of the TLR4 are concatenated and are called Toll/IL‐1R homology (TIR) domains.^46^, ^47^ The TIR domain of TLR4 combines with the TIR domain of MyD88. Meanwhile, MyD88 contains a death domain that interacts with the IRAK's death domain to activate the signalling of the TRAF6/IKKs/NF‐κB axis.^48^ These results showed that the levels of MyD88, TRAF‐6 and IRAK‐1 in human chondrocytes were increased after LPS stimulation, whereas Astilbin pre‐treatment reversed these. Interestingly, these results are consistent with the findings of Chen et al, who found that Astilbin attenuates inflammation in rat glomerular mesangial cells by inhibiting the TLR4/MyD88/NF‐κB pathway.^26^

A DMM‐induced OA mouse model was established in vitro and used to confirm the protective effect of Astilbin on the cartilage in vivo. Mice in the DMM group showed obvious cartilage calcification and erosion. However, treatment with Astilbin reversed this phenomenon, and the OARSI score is consistent with these results. The results indicated that Astilbin has the potential to reduce the development of OA in vivo.

In conclusion, this study demonstrated that Astilbin alleviates LPS‐induced inflammation and ECM catabolism by inhibiting the TLR4/MD‐2 axis and NF‐κB signalling pathway in human OA chondrocytes. In vivo, Astilbin protects the cartilage and delays the progression of OA. This study demonstrates that Astilbin can be considered as a potential therapeutic drug for OA.

## CONFLICT OF INTEREST

The authors declare no conflict of interest.

## AUTHOR CONTRIBUTION

**Shuaibo Sun:** Methodology (equal); Project administration (equal); Writing‐original draft (equal). **Weihui Qi:** Data curation (equal); Software (equal). **Xiaolong Shui:** Writing‐review & editing (equal). **Yanlin Chen:** Data curation (equal); Software (equal). **Xinxian Xu:** Data curation (equal); Software (equal). **Yuezheng Hu:** Formal analysis (equal); Supervision (equal). **Zijian Yan:** Writing‐review & editing (lead). **Weijun Guo:** Writing‐review & editing (lead). **Ping Shang:** Formal analysis (equal); Methodology (equal); Project administration (equal); Writing‐original draft (equal).

## Data Availability

The data used to support the findings of this study are available from the corresponding author upon request.
